# Action Spectrum for Photobleaching of Human Lenses by Short Wavelength Visible Irradiation

**DOI:** 10.1371/journal.pone.0123732

**Published:** 2015-04-17

**Authors:** Line Kessel, Michael Larsen

**Affiliations:** 1 Department of Ophthalmology, Glostrup Hospital, Glostrup, Denmark; 2 Department of Clinical Medicine, Faculty of Health Sciences, University of Copenhagen, Copenhagen, Denmark; Rush University Medical Center, UNITED STATES

## Abstract

**Purpose:**

Cataract is the world-leading cause of blindness. In search for a new treatment of cataract we have found that the yellow discolouration of aged human lenses can be photobleached using a non-invasive, infra-red, femtosecond laser treatment. These results were presented in an earlier PlosOne publication. The objective of the study was to characterize the single-photon photobleaching action spectrum of the aged human lens in vitro.

**Methods:**

Ninety-one human donor lenses were irradiated with continuous wave laser light at 375, 405, 420, 445, 457 or 473 nm. Photobleaching was monitored by photography and transmission measurements.

**Results:**

The action spectrum peaked at 420 nm followed by, in order of decreasing effect, 445, 457, 473, 405 and 375 nm. Younger and less absorbent lenses showed smaller changes than older and more absorbent lenses. There was a dose-dependent increase in lens transmission with increasing laser irradiation.

**Conclusions:**

For a 75 year old lens an effect corresponding to elimination of 15 years or more of optical ageing was obtained. This study of the spectral characteristics and intensity needed to bleach the human lens with single-photon laser effects found an action-spectrum peak at 420 nm tailing gradually off toward longer wavelengths and more steeply toward shorter wavelengths. The results may be used to guide experiments with two-photon bleaching.

## Introduction

In spite of an effective treatment, cataract remains the world-leading cause of poor vision accounting for 20 million cases of bilateral blindness [1]. The high prevalence of blindness in Third-world countries is mainly related to a lack of trained ophthalmic surgeons and operating facilities with the number of ophthalmologists being as low as 1 per 2.6 million in Ethiopia compared to 1 per 20.000 in Europe and North America [2]. Even in the industrialized world, cataract is a significant health care problem and it is expected to increase as the proportion of senior citizens rises [3]. This need cannot only be met by increasing the number of ophthalmologist because the elderly population also has other age-related eye diseases such as age-related macular degeneration and glaucoma that are expected to increase concomitantly and which will also require health care resources [4]. New technology is moving cataract surgery toward increasing technical complexity and higher infrastructure costs [5] but does not reduce the need for skilled ophthalmic surgeons.

The subjective symptoms of cataract range from a subtle impairment such as difficulties reading in the dark to overt blindness. The symptoms of cataract are caused by two main optical features: increased absorption and increased scattering of light. Biochemically, cataract is a protein conformational disorder [6]. Absorption of light is caused by accumulation of chromophores. Scattering is caused by accumulation of large protein aggregates but also by changes in protein-protein interaction due to changes in the tertiary structure of the proteins induced by a variety of degrading mechanisms. Lens chromophores are formed by two main pathways; phototransformation of tryptophan and tryptophan products [7–9] and non-enzymatic glycation of intrinsic lens proteins [10–12].

We have proposed non-invasive photobleaching as a potential means of reducing the need for cataract surgery and shown that in vitro this method can produce pronounced optical effects. This may be clinically relevant for the prevention or early treatment of cataract, a disease of very slow and gradual onset, and can be made non-invasive using short infrared femtosecond laser pulses that can produce two-photon effects that are equivalent to single blue photons in a targeted area deep inside the lens [13]. To determine the action spectrum for photobleaching, the present study investigated single-photon bleaching experiments in the UV-blue wavelength range in preparation for the development of a two-photon instrument for clinical testing.

## Methods

### Human lenses

Intact human donor lenses were placed in an upright position in 5 mm path-length quartz cuvettes. The cuvettes were filled with a neutral saline solution containing 8.00 g/l NaCl, 0.40 g/l KCl, 0.10 g/l Na_2_HPO_4_, 1.00 g/l glucose, and 2.38 g/l Hepes. The solution was buffered to a pH of 7.4 using NaOH. A circular aperture of 2 mm in diameter was placed externally on the cuvette at the centre of the lens on the side of the cuvette facing the anterior lens surface. All transmission measurements and all irradiations were made through this aperture along the optical axis of the lens in an anterior-posterior direction. The study was approved by the Institutional Review Board of the Capital Region of Denmark (H-3-2011-035). The Institutional Review Board waived the need for consent becaused the donor material was anonymous.

### Transmission measurements

The effect of irradiation was evaluated by measuring the transmission of white light through the irradiated area before and at various time points during the irradiation procedure. A broad-band source of white light (DT-Mini-2-GS, Micropack, Ocean Optics, Netherlands) was coupled to an optical fibre, the other end of which was placed in front of the lens. Light transmitted through the lens was collected by an integrating sphere (FOIS-1, Ocean Optics, Netherlands) that was coupled to a spectrometer using an optical fibre (USB4000, Ocean Optics, Netherlands). Transmission was calculated as the ratio between transmitted and incident light after correction for background levels of light as described previously [14]. To correct for fluctuations in the intensity of the light source all measurements were calibrated by setting the transmission from 650 to 700 nm to 100% since transmission at this band of the spectrum changes little with age [15]. For the evaluation of the effect of photobleaching the attention was focused on the transmission of blue light (450–490 nm) since the largest age-related changes in transmission takes place in the blue wavelength region [14]. Blue light lens transmission was evaluated as the area under curve of transmission from 450 to 490 nm.

### Irradiation

Lenses were irradiated using a collimated beam of laser light from either of six different monochromatic, continuous-wave (cw) lasers. The laser beam cross section was kept greater than the aperture on the front side of the cuvette holding the lens to ensure that the entire area of the aperture was irradiated. The lasers used were a 375 nm laser (RGBLase, Fremont, CA, USA) with an output of 6.5 mW, a 10 mW 405 nm laser (World Star Tech, Toronto, Canada), a 5.3 mW 420 nm laser (Prixmatix, Modiin-Ilite, Israel), a 18 mW 445 nm laser (RGBLaser, Fremont, CA, USA), a 13 mW 457 nm laser (Changchun New Industries, Changchun, China) and a 25 mW 473 nm laser (Ningbo Lasever Inc, Ningbo, China). The laser power was measured through the 2 mm aperture using a thermopile detector (Newport 1918-C hand-held optical meter, Newport Corporation, USA).

Statistical calculations were performed using the Sigma Plot software (version 10.0, Systat Software, Inc). The level of significance was set at a p-value of 0.05.

## Results

A total of 91 lenses covering an age range of 26 to 80 years were used for the experiments. Prior to irradiation all lenses had the normal characteristics of an aging lens but no localised opacities. After irradiation, none of the lenses showed any sign of increased colouration, decreased transmission or localised opacities.

**Fig 1:**
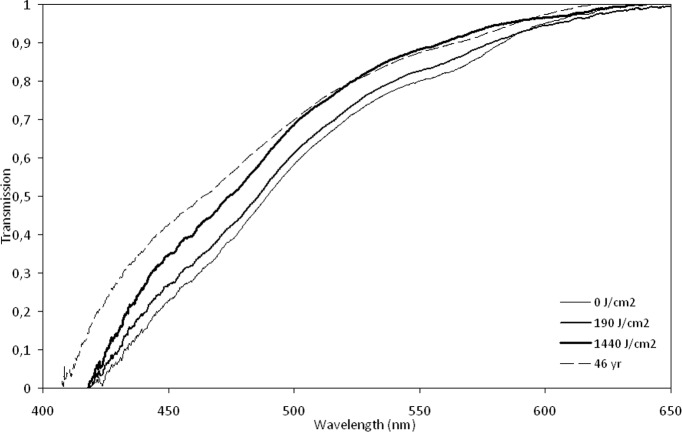
Transmission changes after laser irradiation at 420 nm. The graph demonstrates the changes in transmission measured after irradiation by a 420 nm cw laser for a 68 year old human lens. From a baseline transmission that was lower than a 46-year old non-irradiated reference lens throughout the spectrum, transmission gradually increases to approach that of the younger reference lens after a dose of 1440 J/cm^2^.

**Fig 2:**
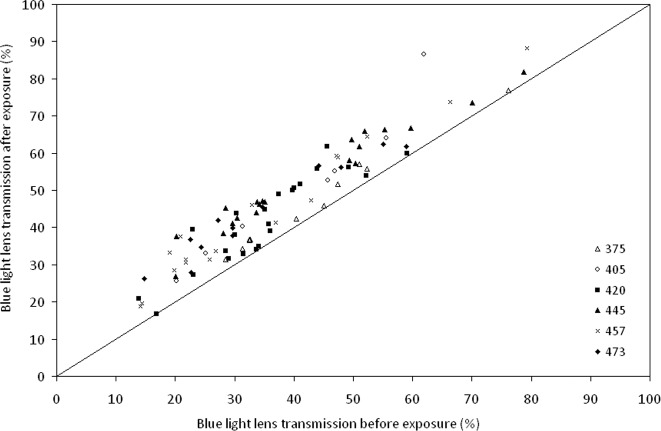
Comparison of blue light transmission before and after laser irradiation. The graph shows the transmission of blue light (450–490 nm) before (x-axis) and after (y-axis) laser irradiation at 375 nm (**△**), 405 nm (○), 420 nm (■), 445 nm (▲), 457 nm (×) and 473 nm (♦) for all 91 lenses included in the experiments.

Laser exposure was followed by improved transmission over nearly the entire width of the visible spectrum, see Fig 1. In the blue light region (450–490 nm), where age-related transmission loss is most prominent, transmission increased after exposure in all lenses. On average transmission increased by 26% and 53 lenses (58%) showed a transmission increase >20%. In 11 lenses (12%) the transmission increase was <5%, see Fig 2.

To examine if the photobleaching effect showed a dose-dependency on the irradiation dose a mathematical function was applied to the result from each single lens. A quadratic polynomial function was found to provide the best fit. The majority of lenses (n = 69; 76%) showed a significant dose-response relation so that transmission increased with increasing irradiation doses, see Table 1. In 20 lenses (23%), no certain dose-response effect was seen even though blue light lens transmission did increase on average 9% after irradiation in these lenses. These 20 lenses were significantly younger (58.6 (17.2) years, mean (SD)) than the lenses that did respond in a dose-response manner to laser exposure (65.5 (9.6), p = 0.02, t-test).

**Table 1:** Dose dependency of irradiation on the transmission of blue light.

	*Age of donors*	*Lenses*	*Dose-dependent*	*y0*	*a*	*b*	*c*	*Adjusted R* ^*2*^	*RMSE*
375 nm	64.2 (36–76)	11	5	0.018(0.008)	0.963(0.020)	0.001(0.000)	0.493(0.065)	0.99	0.011
405 nm	62.9 (46–80)	8	7	-0.083(0.020)	1.193(0.031)	0.001(0.000)	0.584(0.147)	0.97	0.041
420 nm	66.5 (41–77)	23	14	-0.001(0.014)	1.017(0.037)	0.002(0.000)	0.521(0.191)	0.91	0.032
446 nm	59.4 (26–74)	20	17	0.025(0.016)	0.971(0.034)	0.003(0.000)	0.401(0.109)	0.98	0.043
457 nm	62.6 (30–74)	17	14	0.010(0.007)	0.993(0.018)	0.003(0.000)	0.414(0.104)	0.97	0.027
473 nm	68.7 (56–77)	12	12	0.024(0.008)	0.940(0.020)	0.005(0.000)	0.336(0.159)	0.97	0.022

The age (mean with range in parenthesis) of the donors used for the experiments is shown in the second column. The number of lenses used for each wavelength is shown in the third column and the number of lenses showing a significant dose-dependent increase in transmission as a function of increasing irradiation doses is shown in the fourth column.

The following columns show the result of modelling the photobleaching observations to the mathematical function: Trans_post_ = y0 + aTrans_pre_ + bDose^c^. Trans_post_ is the transmission of blue light after irradiation and Trans_pre_ is the transmission of blue light before irradiation. The dose of irradiation was measured in J/cm^2^. y0, a, b and c are constants. The constants are shown as parameter estimates with standard error of estimate in parenthesis. Only lenses showing a dose-dependent photobleaching effect were included in the modelling. RMSE: root mean square error.

**Fig 3:**
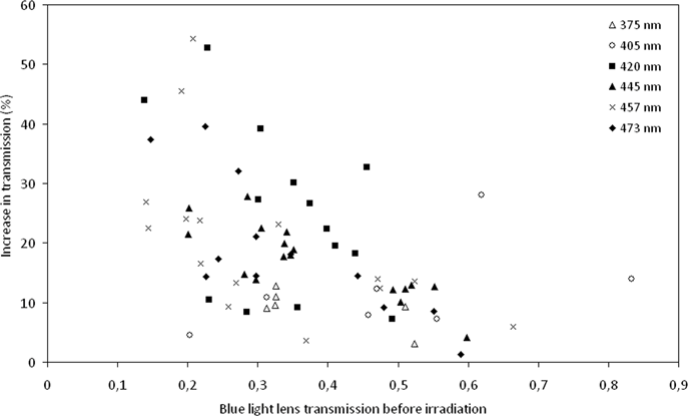
Transmission increase as a function of pre-treatment light absorption. The relationship between transmission of blue light before irradiation with the increase in transmission (in %) after transmission for each of the 6 different irradiation wavelengths used in the experiments. To facilitate comparison between different lenses the effect is normalized to an irradiation dose of 1000 J/cm^2^ for all lenses.

The densely yellow lenses (lower blue light transmission) from older donors showed larger increases in blue light transmission after irradiation than the less yellow lenses of younger donors, see Fig 3.

**Fig 4:**
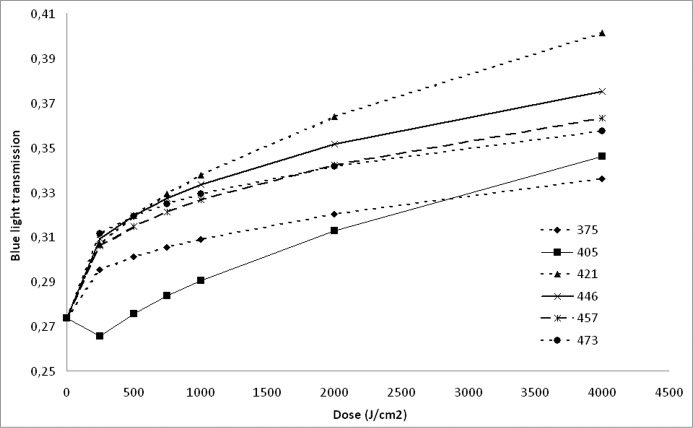
Dose-dependency of photobleaching on the transmission of blue light computed using the mathematical expressions presented in Table 1 for a 75 year old lens for each of the six irradiation wavelengths used.

When evaluated by blue light lens transmission, photobleaching effects were most pronounced at 420 nm and lowest at 375 nm, see Fig 4. The peak of the photobleaching action spectrum at 420 nm was followed, in decreasing order of effect by 445, 457, 473, 405 and 375 nm.

To define a parameter of clinical relevance, the effect of photobleaching was expressed as the change in the apparent age of the lens as judged on the basis of its optical characteristics. For this purpose, transmission of blue light as a function of age in untreated lenses (Trans_blue_) was modelled by Eq 1 (adjusted R^2^ = 0.58, p<0.001):

$$Trans_{blue}=1.017-0.00991\times Age$$ (1)

**Fig 5:**
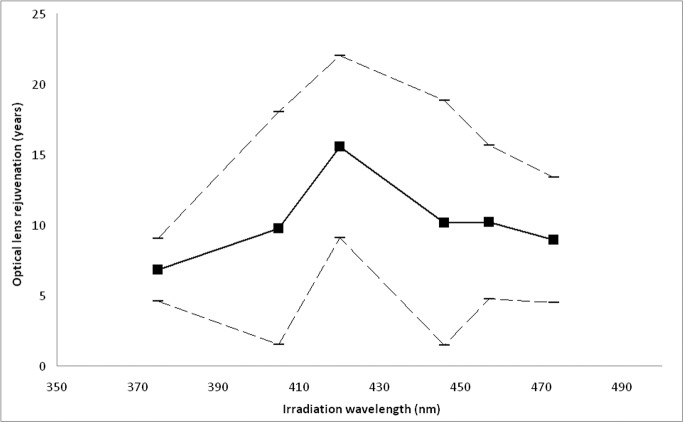
Effect of irradiation wavelength on optical lens rejuvenation for a standard 75 year old lens. The photobleaching effect for each irradiation wavelength was calculated using the formulas presented in Table 1 using an irradiation dose of 4000 J/cm^2^. The photobleaching effect was transformed into a clinically interpretable factor by calculating the apparent lens age after photobleaching using Eq 1. The numbers on the y-axis present the difference between the apparent lens age before and after irradiation. The photobleaching effect is presented as mean value (thick line), upper and lower 95% confidence intervals (thin hatched lines).

To compare photobleaching efficiency between different wavelengths, an apparent lens age was calculated before and after irradiation for a model 75 year old lens using Eq 1. After an irradiation dose of 4000 J/cm^2^ the mean reduction in apparent lens age was 6.8, 9.7, 15.6, 10.1, 10.2 or 9.0 years for 375, 405, 420, 445, 457 or 473 nm, respectively, see also Fig 5.

## Discussion

We examined the effect of short wavelength visible and UVA irradiation of naturally aged human donor lenses and found that the majority of lenses were significantly photobleached after laser irradiation. Visual inspection did not reveal any signs of localized lens opacities in treated lenses. The long term effects of this experimental procedure were not studied in this experiment because of the limited time that lenses can be stored ex vivo before they lose their optical clarity.

Our main outcome was changes in total visible light lens transmission after photobleaching. Cataract is, however, characterised by both loss of transmission and increased light scattering. We did not assess scattering in this study but in a previous study we found that the lenses, that are available for research from organ donors, show very little scattering [16], probably because of easy access to cataract surgery for those patients who have visually significant cataracts and hence increased lens scattering.

The photobleaching action spectrum found in this study did not correspond to the spectral absorption of the lens. The aged human lens absorbs strongly in the ultraviolet [17,18] and short wavelength visible region of the spectrum [14,15] because of an accumulation of chromophores. The effect per unit photonic energy was lowest at 355 and 405 nm and highest at 420 nm. It should be noted, though, that transmission was measured through the entire thickness of the lens. If high absorption confined the photobleaching effect at short wavelengths to the outermost layers of the lens, then that may have limited the measurement of the bleaching effect.

The effects of irradiation with UVB (290–315 nm) and UVA (315–400 nm) have been studied extensively but very little is known about the effect of visible electromagnetic radiation on the lens. The action spectra for phototoxic damage on lens epithelial cells [19] and intact porcine lenses [20] indicate that wavelengths longer than 400 nm are harmless. Irradiation with UVA bleached intact human lenses and reduced their autofluorescence [21,22] but in contrast to homogenates of human lens, no loss of tryptophan or histidine occurred, indicating that the intact lens is relatively resistant to singlet-oxygen mediated photodamage [22]. This is in spite of the high content in the aged lens of photosensitizers capable of producing reactive oxygen species and singlet oxygen [23,24]. The very low oxygen concentration [25,26] in the intact lens may limit its susceptibility to photooxidation.

We have demonstrated that photobleaching has the potential to increase light transmission corresponding to at least 6.8 to 15.6 years of optical aging effects in the human lens. In a hypothetical scenario this could, because of the steep increase in cataract incidence with age, reduce the need for cataract surgery by more than 50% [3]. However, the effects of the photobleaching procedure on the scattering properties of the lens as well as long-term effects of the optical quality of the lens were not assessed in the present study and thus the results can only serve as a stimulus for further research.

Short wavelength irradiation is hazardous to the retina and the safety dose is around 31.5 J/cm^2^ of retinal illumination at 420 nm [27]. Assuming that only the central 6 mm zone of the human lens would need treatment and taking into account the limited transmission of violet light in the aged lens (8.1% in a healthy 70 year old lens) [14], retina-safe laser photolysis treatment of the lens at 420 nm could be made, in theory, by utilization of up to 3900 J/cm^2^ at the anterior surface of the lens, provided that the laser is defocused and spread out over one third of the inside of the globe. We limited our maximum irradiation doses to this presumably retina-safe window. It should be considered, however, that exposure to short wavelengths below the threshold for acute phototoxicity may be a risk factor for age-related macular degeneration (AMD) [28,29]. Therefore, two-photon photo bleaching may be an attractive option as we have previously proposed [13]. The technique requires focused and scanned irradiation rather than the diffuse irradiation used in the present study but it is safer for the cornea and the retina because it uses infrared rather than visible light.

Although the aim of the study was to examine the action spectrum of single-photon photobleaching of the aged lens in preparation for future design of two-photon photobleaching with eye-safe infra-red femtosecond lasers, results from single-photon experiments may not be directly translated into requirements for two- or multiphoton experiments since continuous wave and single-photon effects (as we used in this study) relies on linear absorption and two- or multiphoton effects relies on non-linear absorption [30]. Generally, laser-tissue interactions can be grouped in photochemical, thermal and mechanical effects [31]. In our line of research we aim for photochemical effects since thermal effects would lead to aggregation of lens proteins [32] which in turn causes lens opacities [33] and mechanical effects causes tissue disruption [30]. We did not observe any focal opacities or signs of tissue-disruption in any of the treated lenses.

## Conclusion

The study demonstrated that chromophores of the human lens can be bleached by visible light. Effects of potential clinical value were obtained with irradiation doses that appear to be within the range of what can be safely applied to the intact human eye, but careful preclinical safety studies would have to be made before clinical experiments can be considered. A better approach may be to obtain the photobleaching effects by two-photon processes induced by safer infrared lasers. Many questions concerning the technique of photobleaching as a potential future treatment of cataract remains unanswered. Cataract is optically characterised by decreased transmission and increased scattering of light. The present study addressed the effect of photobleaching on transmission but not on light scattering. The long-term effects of photobleaching are unknown. Nevertheless, results so far are promising and since the need for cataract treatment is increasing, further research into photobleaching as a potential prophylactic or therapeutic remedy for cataract is warranted.
